# Accounting for inequality in dementia prevention programmes

**DOI:** 10.2471/BLT.25.293220

**Published:** 2025-12-10

**Authors:** Timothy Daly, Andrea Slachevsky, Dominic Trépel, Sebastian Walsh, Agustin Ibáñez

**Affiliations:** a Bioethics Program, FLACSO Argentina, Buenos Aires, Argentina.; b Memory and Neuropsychiatric Center, University of Chile, Santiago, Chile.; c Global Brain Health Institute, Trinity College Dublin, Dublin, Ireland.; d Cambridge Public Health, University of Cambridge, Cambridge, England.; e Latin American Brain Health Institute, Universidad Adolfo Ibáñez, Av. Diag. Las Torres 2640, 7941169, Peñalolén, Santiago, Región Metropolitana, Chile.

Social and health inequalities are drivers of disparities in the risk of developing dementia,^1^ a leading and growing contributor to morbidity worldwide. However, most dementia prevention research and policy-making efforts have overlooked these inequalities and have mainly focused on individual and lifestyle behaviours. In this article, we focus on dementia prevention from a multilevel public health perspective, emphasizing structural and social factors as key components for effective intervention.

Dementia is a progressive syndrome marked by cognitive, functional and neuropsychiatric decline, affecting more than 57 million people globally.^1^ Dementia results from a complex interplay between biological factors, including but not limited to genetics, overall health and the environment. The 2024 report of the *Lancet* Commission on dementia prevention, intervention and care showed that 45% of the population attributable fraction of cases worldwide is associated with 14 potentially modifiable risk factors related to education, overall health (physical, mental and social) and air pollution.^1^ The report aims to communicate a clear and accessible message about risk to lay audiences, health workers and policy-makers, presenting risk in a linear and visual manner, detached from the structural factors associated with it, such as socioeconomic status and the built and natural environment in which people live, grow and work. The decontextualized framing of risk has dominated research and policy over the past 10 years,^2^^,^^3^ focusing on the therapeutic strategy of conscious individual behaviour change to achieve dementia risk reduction.

However, this approach presents a paradox. Health inequalities exist largely because disadvantaged individuals face physical and socioeconomic living conditions that pose a higher risk to their health, and they have limited opportunities to change the structural factors behind these conditions.^4^ Resources required for sustained behavioural change are unevenly distributed across populations, meaning that interventions relying solely on individual financial, social and motivational resources inherently widen health inequalities.^3^ This pattern also applies to dementia prevention, underscoring the necessity of structural approaches rather than exclusively individual ones.^2^^,^^3^

In interventions targeting individual behaviours, better-resourced groups benefit more, which can lead to ongoing research and policy unintentionally widening health disparities.^3^ In response, a growing number of researchers have adopted a life-course approach to dementia prevention to understand the accumulation of risk and protective factors across the lifetime.^1^ Individuals from underserved backgrounds often face multiple simultaneous and longitudinal barriers such as limited education, economic constraints and adverse environmental conditions worsening their health and restricting their benefit from behaviour-focused interventions. In other words, the individual effort required to stay healthy differs between different socioeconomic contexts: staying healthy demands more effort in a disadvantaged environment than in an advantaged one.^4^

The most robust evidence from meta-analyses for social determinants of dementia exists for less education (5% of population attributable fraction) and air pollution (3%).^1^ A recent risk model estimation found that early life poverty (2%), mid-life income inequality (2%) and late-life wealth shocks (14%) were independent risk factors for dementia.^5^ These findings align with the *Lancet* report’s observation that many risk factors for dementia cluster around socioeconomic inequalities and deprivation, air pollution, unhealthy lifestyles and lower educational attainment.^1^

Thus, while well intentioned, behaviour-focused interventions neglect the broader social, economic and environmental contexts. Dementia risk accumulates from a complex interplay of factors over decades, and an individual-focused intervention typically addresses health behaviours without tackling the cumulative impact of risk factors. While individual interventions (like promoting exercise) can be beneficial, their effects may be small and incremental, especially if underlying conditions (like widespread poverty) remain unchanged.^3^^,^^4^

Addressing multiple risk factors simultaneously places greater demand on individuals, requiring numerous concurrent behaviour changes. The burden is currently on people, not the health systems, because behaviour-based prevention requires individuals to change many things at once. Consequently, efforts that focus on personal responsibility are more likely to favour populations with greater resources, inadvertently exacerbating existing inequalities in dementia risk and outcomes.^3^

We argue that incorporating population-level measures is necessary to correct the current bias towards individual-focused initiatives.^3^ Doing so involves shifting emphasis to also implementing structural changes that enable those behaviours, for example policies that create healthy environments. This approach complements personal interventions, because it encourages individual behaviour change by providing a supportive context for it. Our position also aligns with the World Health Organization’s (WHO) *A blueprint for dementia research*,^6^ particularly Strategic Goal 14, which addresses diverse social, environmental and biological risk factors, and Goal 15, which calls for robust evidence supporting both individual- and population-level interventions.

The evidence for inclusion of population-level approaches to dementia prevention is threefold. First, compelling evidence exists, particularly from countries in the WHO Region of the Americas, demonstrating that structural factors increase the ageing burden from healthy ageing^7^ to accelerated brain ageing.^8^ Multiple exposure factors widen the gap between chronological age and accelerated biological ageing.^9^ Specifically, physical exposures (pollution), social determinants (socioeconomic status, inequalities, migration) and psychosocial stressors (democratic instability) exhibit compounded risk effects that accelerate ageing clocks across global populations.^8^ These effects are particularly severe in underserved regions, such as the WHO African Region.^9^

Second, the biological effects observed in individuals reflect the influence of population-level factors such as socioeconomic and environmental conditions. Unsurprisingly, at the population level, the effects of broader interventions on dementia prevention can be measured. Age-adjusted incidence of dementia in high-income countries has decreased by up to 13% (95% confidence interval: 7–19) per decade^1^ in post-World War II birth cohorts, corresponding to reductions in health inequalities rather than specific measures to address individual behaviour.^10^ These results are not replicated in low- and middle-income countries, where exposure to factors of unhealthy ageing remains higher.^8^^,^^9^ Public health strategies to reduce dementia risk worldwide should therefore also address structural determinants of health, including legal, physical and socioeconomic environments.^1^ This structural focus prevents inadvertently stigmatizing disadvantaged populations.^4^ Governments and policy-makers can draw on many international structural interventions from the 20th century, such as welfare programmes in Nordic countries, United States of America’s Great Society initiatives, Germany’s post-reunification health-care reforms and Brazil’s democratization-linked social welfare expansions.^10^ These ambitious policies can contribute to people living longer in good health and to protecting them from dementia in older age;^1^ they also provide conditions for equitable behavioural strategies by establishing supportive environments that empower sustained healthy behaviours.^3^

Economic studies demonstrate that excess health-care costs arise in the decade before, and immediately after, a formal dementia diagnosis,^11^ and that most of the costs associated with dementia are associated with multimorbidities such as cardiovascular disease and frailty.^12^ Cost analyses, conducted across the world, indicate that modifying social settings that influence smoking behaviour and the fiscal conditions governing tobacco purchase, improving food and physical environments, enhancing educational access and enforcing bicycle helmet legislation could yield significant health-care cost savings while lowering population-level dementia risks.^13^^,^^14^ These findings suggest that multisectoral actions to improve overall health are necessary to protect the ageing brain.

Third, because of the brain’s unique position at the interface between the body and mind, measures that improve brain health will also enhance bodily and mental health, ultimately leading to better outcomes for other diseases, and vice versa.^15^ We emphasize the need for a rights-based or brain health-for-all approach, based on collective action, rather than a responsibility-driven framework of individual behaviour change.^15^

The focus on individual actions rather than societal change for dementia prevention is problematic for two main reasons. First, unlike other conditions such as obesity prevention in children to which broader public health approaches have been applied, dementia research and policy-making have been heavily biased towards individual-level approaches in contemporary history.^2^^,^^3^ Second, dementia is associated with the accumulation of poor overall health and mixed brain pathologies over the lifetime, suggesting that the broadest action possible should be taken to reduce risk and overall costs associated with dementia. Thus, a range of policy entry points are needed to reduce dementia risk, including ambitious public health action against structural and more proximate determinants.

We thus propose enhancing existing dementia prevention frameworks by integrating robust, population-level structural interventions whose scale and intensity can be adapted to the level of disadvantage^3^^,^^4^ to achieve greater health equity. We also recognize the necessity of population-level and individual-level action to achieve equitable, effective and sustainable health outcomes. Emerging evidence suggests that changes to the physical environment, financial interventions and programmes aimed to change the social environment could be cost-effective in reducing modifiable dementia risk in both high- and low- and middle-income countries.^14^ Future research should prioritize building the evidence base for structural interventions in low- and middle-income countries.

Our approach complements the section of the 2024 *Lancet* report on dementia that mentions the need for a focus on socioeconomic factors,^1^ WHO’s founding mission of health for all^15^ and WHO’s *A blueprint for dementia research*.^6^ Approaches in both policy and research that address dementia risk from a whole-population perspective are needed yet understudied, but are now emerging. We propose to bolster them with actionable recommendations for policy, research and advocacy to put action against structural inequalities at the centre of priorities for achieving effective and equitable dementia risk reduction (Box 1).

Box 1 **Actionable recommendations for policy, research and advocacy for brain health equity**

**Create policies to reduce brain health inequities**

Ensure that policy is rooted in population-level understanding of brain health^2^^,^^3^ that:

- is based on the best available scientific evidence;^4^
- responds to local brain health needs; and
- is monitored to avoid the creation of new inequalities.

Focus on primordial and primary prevention^6^ (that is, changing physical, social and legal environments)^1^^,^^12^^,^^13^ so that they are more conducive to lifelong brain health by reducing risk factor development or maintenance, by:

- reducing excess alcohol use through minimum unit pricing;
- reformulating food products to reduce cardiometabolic risk;
- reducing air pollution through low-emission zones;
- raising cigarette prices and targeting social smoking environments;
- making bicycle helmets mandatory;
- changing the physical environment to increase physical activity; and
- using other WHO priority interventions for noncommunicable diseases.

Reduce the impact of unfair wealth distribution on brain health^5^ through:

- investing in early-life safety nets to protect the developing brain from poverty;
- promoting equity-based approaches to reduce income inequality; and
- ensuring robust pension systems to protect people from late-life wealth shocks.

**Build the evidence base**

Build the evidence base for structural interventions in low- and middle-income countries.^7^^–^^9^

Communicate the need for structural interventions to policy-makers and the public.^1^

Conduct qualitative research to determine and respond to local needs.^15^

**Support the right to health**

Advocate for the right to education for all children.^15^

Advocate for the right to universal health coverage.^8^^,^^15^

Support UN and WHO initiatives on the right to a healthy environment.^15^

UN: United Nations; WHO: World Health Organization.Notes: recommendations are based on the authors’ review of the cited references. Readers can also consult the Evidence Synthesis Hub on the International Research Network on Dementia Prevention website, available at: https://coghealth.net.au/evidence-synthesis-hub/, which brings together systematic reviews and/or meta-analyses for a range of risk factors for cognitive decline and dementia.

Promoting brain health equity requires a coherent shift from the individual-level towards structural- and population-level actions that embed prevention across the life course. Policies must be evidence-based and tailored to local needs, and governments should reshape physical, social and legal environments to reduce the development and persistence of risk factors. Economic protections such as early-life safety nets and robust pension schemes are required to address the unequal distribution of opportunities for healthy ageing. Research priorities include building a stronger evidence base on structural interventions, and conducting qualitative work that captures local experiences and highlights the need for action upstream of individual behaviours. Finally, advocacy efforts centred on universal rights to education, health care and a healthy environment may provide the normative foundation needed to sustain political commitment. Collectively, these recommendations position structural determinants at the centre of global dementia prevention efforts, offering a practical roadmap for achieving fairer, more effective and population-wide gains in brain health.
